# Synthetic Peptides as Potential Antigens for Cutaneous Leishmaniosis Diagnosis

**DOI:** 10.1155/2017/5871043

**Published:** 2017-03-07

**Authors:** Juliana Seger Link, Silvana Maria Alban, Carlos Ricardo Soccol, Gilberto Vinicius Melo Pereira, Vanete Thomaz Soccol

**Affiliations:** ^1^Basic Pathology Department, Federal University of Paraná, Curitiba, PR, Brazil; ^2^Department of Bioprocess Engineering and Biotechnology, Federal University of Paraná, Curitiba, PR, Brazil

## Abstract

This work's goal was to research new candidate antigens for cutaneous leishmaniosis (CL). In order to reach the goal, we used random peptide phage display libraries screened using antibodies from* Leishmania braziliensis* patients. After selection, three peptides (P1, P2, and P3) were synthesized using Fmoc chemistry. The peptides individually or a mixture of them (MIX) was subsequently emulsified in complete and incomplete Freund's adjuvant and injected subcutaneously in golden hamsters. Sera from the hamsters administered with P1 presented antibodies that recognized proteins between 76 and 150 kDa from* L. braziliensis*. Sera from hamsters which had peptides P2 and P3, as well as the MIX, administered presented antibodies that recognized proteins between 52 and 76 kDa of* L. braziliensis*. The research on the similarity of the peptides' sequences in protein databases showed that they match a 63 kDa glycoprotein. The three peptides and the MIX were recognized by the sera from CL patients by immunoassay approach (ELISA). The peptides' MIX showed the best performance (79% sensitivity) followed by the P1 (72% sensitivity), and the AS presented 91% sensitivity. These results show a new route for discovering molecules for diagnosis or for immunoprotection against leishmaniosis.

## 1. Introduction

Leishmaniosis is a disease with two principal manifestations: visceral and cutaneous form. These are among the major neglected parasitic diseases that are reemerging and affect about 350 million people worldwide [1]. The disease is caused by the protozoa of the genus* Leishmania* Ross, 1903. The parasites infect humans in the Americas, Africa, Asia, and Europe. In the New World, the cutaneous form of the disease is caused by different etiological agents:* Leishmania (V.) braziliensis, L. (V.) peruviana, L. (V.) guyanensis, L. (V.) panamensis, L. (V.) naiffi, L. (V.) lainsoni, L. (V.) shawi, L. (Leishmania) amazonensis*, and* L. (L.) mexicana*. In Latin America, the widest distribution is presented by* L. braziliensis *and* L. amazonensis.* Visceral leishmaniosis (VL) is caused by* L. infantum (*synonym:* L. chagasi)*, which is more severe and can cause death in the absence of correct diagnosis and early treatment [2].

Advances have been made regarding the diagnosis and prevention of cutaneous leishmaniosis (CL) over the past decade. Moreover, there is no single method that can be adopted as the gold standard [1]. Generally, the combination of two or more indirect techniques is needed to perform an accurate diagnosis [3–5]. The CL diagnosis is frequently based on clinical and epidemiological data associated with laboratory tests. Several laboratorial methods are applied for detecting antibodies and identifying the parasite* Leishmania*. Parasitological methods consist of isolating the parasite from skin ulcers (for cutaneous forms) or from bone marrow or lymph nodes (for visceral forms). Material may be examined while fresh or it can be inoculated in culture medium for multiplication of the promastigotes. Nevertheless, the parasitological method is an invasive practice and demands expertise. Another possibility is the use of techniques researching deoxyribonucleic acid (DNA) of the parasite. However, studies have indicated that parasitaemia may be episodic and may lead to a low number of parasites at the time of collection and hence nondetection by polymerase chain reaction (PCR). In addition, molecular techniques require specific devices, and not all laboratories have such highly complex equipment [6–8]. When these approaches fail, immunological tests are used to provide indirect parameters for the diagnosis. The delayed hypersensitivity skin testing is used for the cutaneous form of leishmaniosis [9]. The tests can detect infection in a few weeks and evaluate cellular immunity. Serological tests that detect antibodies (humoral immune response) are also useful [6–8]. However, immunological tests using promastigotes or soluble protein as a sensitizing antigen source could limit test specificity [4]. The ELISA technique is an alternative method to diagnose cutaneous leishmaniasis (CL). It presents easy execution as an advantage and is able to analyze a great number of samples simultaneously. New antigens that are more specific and sensitive are necessary and could be obtained with the usage of new technology such as recombinant proteins or phage display.

Given this context, the search for purified antigens, with high sensitivity and safety, for the immunological diagnosis and prevention of CL is essential. In recent decades, many molecules have been described as candidate antigens for leishmaniosis serodiagnosis, including some that are purified molecules or soluble fractions of the parasite. In addition, antigens produced by genetic engineering or phage display have been investigated and validated for research on an antibody against* Leishmania* [10–14], especially for the visceral form [15, 16]. However, these antigens still require adequation regarding their sensitivity and specificity values for use in diagnosis and immunoprotection.

Phage display is being used to discover molecules for diagnosis and research in the selection of antigens to be used for the ELISA tests. The immunoscreening of phage-displayed peptide libraries represents an alternative when searching for new antigenic targets. The peptide or protein expressed on the surface of each phage particle can be selected for binding to the target molecules by an affinity selection process called biopanning [17]. Our group has successfully used this technique to identify the epitopes or mimotopes of different pathogens [18–21]. The technology also enables the selection of antigens useful in vaccine production given that there is no effective treatment for numerous diseases [22–24].

This study aimed to select and evaluate synthetic peptides obtained by phage display that can be used in cutaneous leishmaniosis serological diagnosis.

## 2. Materials and Methods

### 2.1. Preparation of* Leishmania *Antigens

The strains of* Leishmania* species were obtained from the cryobank of the Biotechnology Laboratory of the Federal University of Paraná (UFPR) and had previously been characterized by isoenzyme analysis. Antigen from the promastigote culture of* L. braziliensis* (MHOM/BR/94/M2903) was prepared as described by Szargiki et al. [3]. Briefly, cultured parasites were washed with saline 0.9, 0.3, and 0.9% and with phosphate-buffered saline (PBS), pH 7.2, respectively, resuspended in distilled water, and lysed by the freeze/thaw method, followed by sonication. The resulting product was centrifuged at 14,000*g* for 30 min at 4°C. The supernatant was filtered (in a 0.22 *μ*m filter) and recovered, and it featured the soluble antigen (SA). The protein content of the SA was determined by the Lowry method [25]. Aliquots of the antigen were kept at −80°C until they were used.

### 2.2. Patients and Groups

Blood samples were collected from patients diagnosed with clinical CL caused by* L. braziliensis. *This is the main parasite that causes the cutaneous disease in this state of Brazil according to Szargiki et al. [3] and Ribas-Silva et al. [26]. Volunteers came from the Federal University of Paraná's Hospital or several regional departments of health in the same state. The sera obtained were tested by ELISA using the SAs of* L. braziliensis* as described by Szargiki et al. [3]. For parasitological diagnosis, smears from the lesion were taken, stained by May-Grunwald-Giemsa, and observed under an optical microscope (1000x) or skin biopsies of the lesion were ground and inoculated into Tobbie and Evans media, incubated at 24°C, and examined and subcultured every week [3]. As the negative control, serum samples were collected from thirty-seven patients who had no history of leishmaniosis or Chagas disease and no contact with patients infected with these diseases. To point out putative cross-reactivity, sera from patients suffering from other infectious diseases were included in the present study, namely, 10 patients with Chagas disease, 10 patients with leprosy, and 10 patients with tuberculosis. The study was approved by the local research ethics committee (Protocol 107/11-UP).

### 2.3. Anti-*L. braziliensis* Immunoglobulins

Immunoglobulins G (IgGs) of the sera from patients with positive ELISA tests were obtained by precipitation with ammonium sulfate followed by chromatography using protein G-agarose [27]. Anti-*L. braziliensis* IgGs were recovered from immunoblots. For that,* L. braziliensis* SAs were resolved by 15% sodium dodecyl sulphate polyacrylamide gel electrophoresis (SDS-PAGE) [3] and transferred to a polyvinylidene difluoride (PVDF) membrane that was blocked with PBS (pH 7.4) with 0.3% Tween 20 (0.3% PBST), washed with 0.05% PBST (PBS + 0.05% Tween 20), and incubated with IgGs in 0.05% PBST. After washing, IgGs' binding antigens immobilized in the membrane were eluted with 0.1 M glycine and 0.15 M NaCl at pH 2.8 at room temperature for 30 min. Anti-*L. braziliensis* IgGs were dialyzed against PBS after neutralization with 1 M Tris-HCl (pH 9.0), and the protein concentration was determined by the Bradford method [28].

### 2.4. Phage Display

Four rounds of biopanning were performed by incubating four phage display random libraries, obtained from J. Scott (Simon Fraser University, Canada), which expressed 8-mer (LX_4_), 12-mer (LX_8_), 15-mer (X_15_), and 17-mer (X_8_CX_8_) peptides [29], with anti-*L. braziliensis* IgGs. All steps were done according to Alban et al. [19, 20]. Initially, two immunotubes (Nunc, Roskilde, Denmark) were coated with 10 *μ*g of protein G (Sigma-Aldrich, St. Louis, MI, USA) diluted in Tris-buffered saline (TBS; 50 mM Tris, 150 mM NaCl, pH 7.5) and incubated overnight at 4°C. The immunotubes were washed with 0.05% Tris-buffered saline TBST (TBS-0.05% Tween 20), blocked with 3% bovine serum albumin (BSA) in 0.05% TBST for 1 h at 37°C, and washed again. In immunotube one, the incubation was done with 5 × 10^12^ phages from each library overnight at 4°C. In immunotube two, 5 *μ*g/mL of anti-*L. braziliensis* IgG was used, and incubation overnight at 4°C was done. After the incubation period, the supernatant of immunotube two was discarded, and the supernatant of immunotube one was added to immunotube two, where it remained under incubation overnight at 4°C. After washing, the bound phages were eluted with 0.1 M glycine (pH 2.2) and 1 mg/mL BSA. After neutralization with 2 M Tris-HCl, pH 9.0, the eluted phages were amplified by infecting* Escherichia coli* K91 cells. In the second panning, 2.5 *μ*g/mL of anti-*L. braziliensis* was used for coating and 1.5 *μ*g/mL for the remaining ones. After four rounds, phage clones were isolated and screened by ELISA [19]. Briefly, microtiter plates were coated with 0.5 *μ*g/mL anti-phage antibodies (Sigma-Aldrich, St. Louis, MI, USA), in 100 mM NaHCO_3_, pH 8.6, overnight at 4°C. The bacterial supernatant containing phage particles was diluted at 1 : 2 in 2% casein and PBS (pH 7.4) and was added to each well. The plate was incubated for 1 h at 37°C, washed, and incubated for 1 h at 37°C with a patient serum pool that was positive based on ELISA, with* L. braziliensis* SAs diluted at 1 : 100 in the incubation buffer (0.25% casein in 0.05% PBST). After washing, the reaction was detected using an anti-human IgG (Fc-specific) peroxidase antibody. As a negative control, the supernatant of the* E. coli* K91 culture was used.

### 2.5. Peptide Sequencing and Synthesis

The most reactive clones in ELISA tests (those with absorbance at least twice as high as the negatives control) were selected for DNA sequencing and for the subsequent identification of the amino acid sequences inserted in the phages. Phage genomic DNA was extracted with QIAprep Spin Miniprep Kit (Qiagen). Peptide sequences of the phage were determined by BigDye Terminator v3.1 Cycle Sequencing Kit (Applied Biosystems, Foster City, CA, USA) using the reverse primer 5′-GCT GCA TCT TTT AGC AGC-3′. The peptide sequences were analyzed for similarity to protein sequences from* L. braziliensis* or* Leishmania (Viannia) *sp. using the BLAST program.

Syntheses were performed according to standard protocol by using solid-phase 9-fluorenylmethoxycarbonyl (Fmoc) chemistry with a ResPep SL automated peptide synthesizer (Intavis Bioanalytical Instruments, Nattermannallee, Germany). The peptides were lyophilized, and their masses were confirmed by mass spectrometry using a MALDI-TOF/TOF Autoflex instrument (Bruker Daltonics, Bremen, Germany) and flexAnalysis software (Bruker Daltonics, Bremen, Germany).

### 2.6. Reactivity of Anti-Peptides' Antibodies against* L. braziliensis* Antigens

Peptides containing additional cysteine residue on the C-terminal end were conjugated to mariculture keyhole limpet hemocyanin (mcKLH) using the Imject Maleimide-Activated mcKLH Spin Kit (Pierce, Rockford, IL, USA). Four-week-old female golden Syrian hamsters (*Mesocricetus auratus*) were immunized with individual peptides (P1, P2, and P3) or with a MIX of them. Six groups, with ten hamsters for each antigen, were inoculated intradermally (ID), in the back foot with KLH peptide (20 *μ*g for each one) dissolved in a saline solution (0.9% NaCl). For the first immunization, the antigens were emulsified in complete Freund's adjuvant. For the subsequent immunization, we used incomplete Freund's adjuvant. Three boosters were administered at 30-day intervals. As the control, a group received Freund's adjuvant alone under the same conditions. Nonimmune serum was used as the negative control, and immune serum was obtained after seven days of the final immunization. By the end of this period, the animals' blood was collected as well as serum obtained for detection of anti-peptide specific antibodies. The study was approved by research ethics committee (Protocol 26/2012-CEUA-UP).

To assess the production of anti-peptide antibodies, microtiter plates were coated with 2 *μ*g/mL of peptide diluted in 0.05 M carbonate buffer solution, pH 9.6, and incubated overnight at 4°C. After blocking with 2% casein in PBS (pH 7.4), the hamster pool sera diluted at 1 : 50 in the incubation buffer were added to the wells, and the plates were incubated for 1 h at 37°C. After washing, the detection of the reaction was performed using the anti-hamster IgG (whole molecule) peroxidase antibody (Sigma-Aldrich, St. Louis, MI, USA). Western blot assay was performed to evaluate whether the anti-peptide antibodies produced in the hamsters recognized* L. braziliensis* antigens. To achieve this, SAs of* L. braziliensis* (100 *μ*g) were electrophoresed on 15% polyacrylamide gel and transferred to a PVDF membrane, which, after blocking (0.3% PBST), was incubated with hamster anti-peptide pool sera diluted at 1 : 100 in an incubation buffer (3% BSA in 0.05% PBST). The reactivity was detected using anti-hamster IgG (whole molecule) peroxidase antibody at 100 ng/mL in an incubation buffer and revealed with 0.07% (p/v) of DAB (3,3′-diaminobenzidine tetrahydrochloride) in 50 mM Tris, 0.15 M NaCl (pH 7.6), and 0.08% (v/v) H_2_O_2_.

All procedures involving the animals were consistent with the recommendations laid out in the* Guide for the Care and Use of Laboratory Animals* of the Brazilian National Council of Animal Experimentation (http://www.cobea.org.br).

### 2.7. Evaluation of the Potential Diagnostics of Synthetic Peptide

To evaluate the antigenicity of peptides, ELISA procedures were optimized (data not shown). After determining the best condition, microtiter plates were coated with 100 *μ*L of each peptide or with the mixture (MIX) at 5 *μ*g/mL in 0.05 M carbonate buffer (pH 9.6). The plate was incubated overnight at 4°C. After washing with a solution containing 0.9% NaCl with 0.05% Tween 20, the plates were blocked with Protein-Free Blocking Buffer (Thermo Fisher Scientific) for 1 h at 37°C. Then, the plates were washed and incubated with the human sera diluted at 1 : 50 in phosphate-buffered saline (PBS) solution at pH 7.4 containing 0.1% BSA. The plates were kept at 37°C for 1 h, and later they were washed and incubated with anti-human IgG (Fc-specific) biotin antibodies at 0.25 *μ*g/mL in 1% BSA, PBS pH 7.4 for 1 h at 37°C. The plates were washed and incubated with 1 *μ*g/mL of NeutrAvidin-peroxidase at 12.5 ng/mL with 1% BSA diluted in PBS pH 7.4, for 1 h at 37°C. A cutoff point for optimal sensitivity and specificity for the ELISA tests was determined using the Receiver Operating Characteristic (ROC) curve analysis, as described by Metz [30] and Zweig and Campbell [31]. The ROC curve enables the evaluation of the overall accuracy through the area under the ROC curve (Area under Curve, AUC) and the study of sensitivity and specificity with various cutoff points. The information generated subsidizes the identification of the optimal cut point. The curve analysis was performed using MedCalc 13.2.0 (MedCalc Software, Mariakerke, Belgium).

## 3. Results

### 3.1. ELISA Using Soluble Antigens (SAs) of* L. braziliensis *and Screening of Phage-Displayed Peptide Libraries against Anti-*L. braziliensis* IgG

Of the 82 serum samples obtained from patients with CL, 57 were positive against the SAs of* L. braziliensis*. Thus, the CL patients were divided into two groups: Group 1 (G1) consisted of 25 CL patients with positive culture and indirect ELISA test positive using SAs antigen and Group 2 (G2) consisted of 57 CL patients with clinical diagnosis and indirect ELISA test positive for SAs antigen. Anti-*L. braziliensis* immunoglobulins G obtained in Group 2 were precipitated, purified, and incubated with phages that displayed peptide libraries. For each selection round, enrichment was monitored for antibody-specific phage by calculating the percentage of the phage that was bounded. A progressive increase in the phage recovery after each selection round was observed, indicating that specific enrichment of anti-*Leishmania*-binding phages occurred (Table 1).

### 3.2. Peptide Sequences

The fourth selection cycle was chosen to infect a culture of* E. coli* and obtain isolated colonies on plates containing LB medium. Each clone was tested by ELISA using the culture supernatants and sera from patients with high absorbance (tested initially against soluble antigens (SAs) of* L. braziliensis* in ELISA tests presenting absorbance above 0.5). Thus, 428 clones were tested and only 36 of these were reactive against the sera of patients with cutaneous leishmaniosis and three different peptides sequences were identified.

The peptides sequences were compared with others deposited in the GenBank to verify homology to proteins of* L. braziliensis*. The analysis between the peptide sequences and* L. braziliensis* proteins showed that they are like a 63 kDa glycoprotein of* Leishmania *(Table 2). Bold letters denote the consensus sequences between the mimotopes and the* Leishmania* proteins sequence from GenBank.

### 3.3. Immunogenicity of Peptides In Vivo and Antibody Capacity to Recognize* Leishmania*

**Figure 1:**
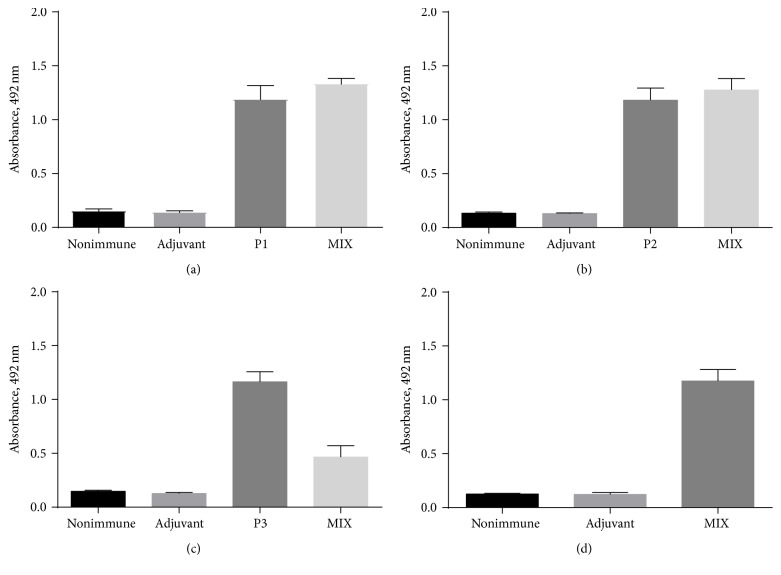
Reactivity of peptides with sera from immunized hamsters by ELISA. P1 peptide (a), P2 peptide (b), P3 peptide (c), and MIX of P1, P2, and P3 (d) were tested with anti-peptide serum P1 (P1), anti-peptide serum P2 (P2), anti-peptide serum P3 (P3), anti-peptide serum MIX (MIX), nonimmune serum, and serum against Freund's adjuvant. ELISA plates were coated with peptide (2 *μ*g/mL) and incubated with hamster serum diluted at 1 : 50 (pool of sera with ten animals in each group). The detection reaction was conducted with anti-hamster IgG (whole molecule) peroxidase antibody (200 ng/mL).

**Figure 2:**
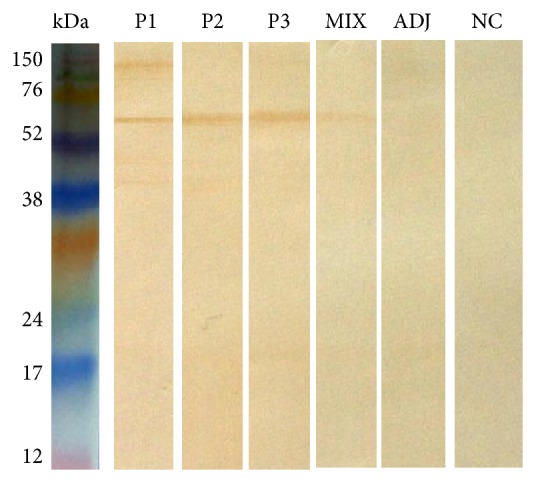
Anti-peptide antibody reactivity to* L. braziliensis* antigens by western blot. Forty micrograms of* L. braziliensis* antigen was separated by 15% SDS-PAGE and then, using western blot, was reacted with anti-peptide serum P1 (lane 1), anti-peptide serum P2 (lane 2), anti-peptide serum P3, anti-peptide serum MIX, sera against Freund's adjuvant (lane 5), and NC = negative control → nonimmune serum (lane 6). Sera from the immunized hamster were tested at a 1 : 100 dilution. The detection reaction was performed with anti-hamster IgG (whole molecule) antibody conjugated to peroxidase (100 *μ*g/mL).

To evaluate the presence of anti-peptide antibodies in hamsters, sera from immunized animals with peptides were assessed by ELISA (Figure 1). All animals that were immunized with the peptide showed reactivity toward peptides 1, 2, and 3 alone and against the pool of peptides. Nonimmunized hamsters and those that were only given the adjuvant did not produce antibodies against the peptides. Anti-peptide antibodies were reactive to the SA of* L. braziliensis *in evaluation in vitro by western blot (Figure 2).

### 3.4. Performance of Peptides for Serodiagnosis of Cutaneous Leishmaniosis

**Figure 3:**
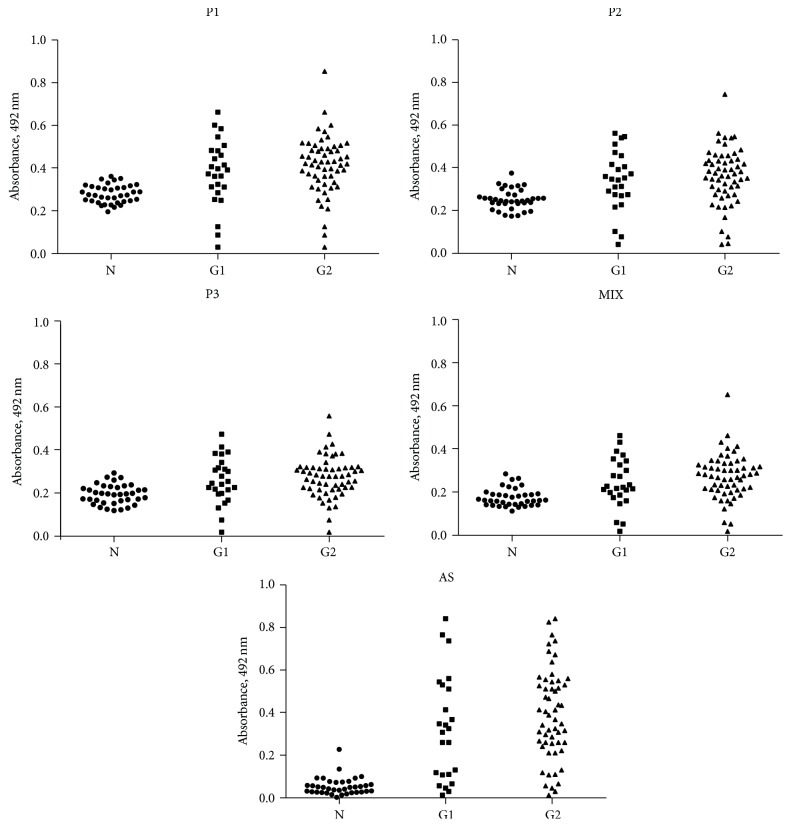
Reactivity of peptides P1, P2, P3, MIX, and SA against sera from CL patients (G1: Group 1, G2: Group 2) and negative control (N).

The three individual peptides and the MIX of them were immunoreactive against the sera of patients in both patients groups G1 and G2. ELISA plates were coated with 5 *μ*g/mL of individual peptide 1, 2, or 3 or MIX (equal concentrations of the three peptides totaling 5 *μ*g/mL) and incubated with human serum diluted at 1 : 50. The detection was performed with anti-human IgG (Fc-specific) biotin antibody and NeutrAvidin-peroxidase. For the test with SA, the plates were coated with 0.5 *μ*g/mL of SA and incubated with a 1 : 100 diluted human serum. The detection was performed with anti-human IgG (Fc-specific) peroxidase. The horizontal line represents the cutoff (provided by the ROC curve). The geometric shapes represent individual results (Figure 3).

**Figure 4:**
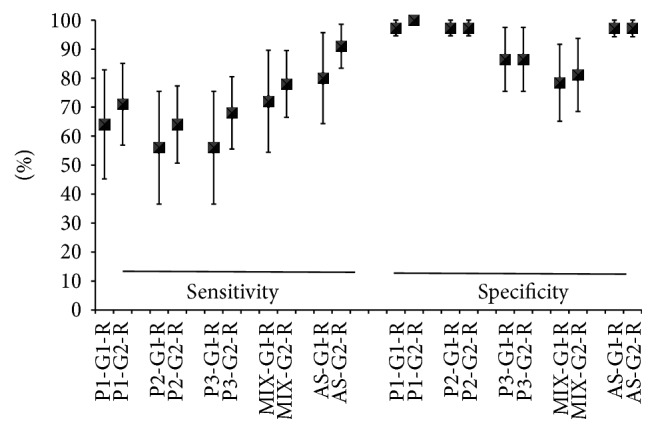
Performance of ELISA test with synthetic peptides and sera of patients with cutaneous leishmaniosis. P1: peptide 1; P2: peptide 2; P3: peptide 3, MIX: P1 + P2 + P3; G1: Group 1 (patients with clinical cutaneous leishmaniosis and parasites isolated); G2: Group 2 (patients with clinical cutaneous leishmaniosis and serological diagnosis positive); SA: soluble antigen.

The MIX of peptides showed 79% sensitivity and P1 showed 72%. The specificities of antigens ranged from 78 to 100%, demonstrating the ability to correctly diagnose the healthy individuals (Figure 4).

For the patients of the G1, when comparing antigens P1, P2, and P3 and the MIX of them with AS, a significant difference was found between MIX and AS (*p* = 0.045). For the patients of the G2, comparing antigens P1, P2, and P3 and MIX with AS, a significant difference was found between P3 and AS (*p* < 0.001) and between MIX and AS (*p* < 0.001). In both groups, no difference was found between P1 and AS and between P2 and AS. In summary, peptides P1 and P2 are not significantly different from AS and may present similar results in the diagnosis of CL.

The use of soluble antigen (SA) in serum of patients with Chagas' disease showed higher cross-reactivity (6/10). For P1, cross-reactivity was observed with Chagas disease (Table 3).

## 4. Discussion

Phage-displayed peptides are called mimotopes because they are not homologue sequences to the antigen. However, they can induce antibodies that recognize the mimotope and the original antigen owing to conformational similarities between them [18]. Our group found evidence that supports phage-displayed peptides as promising biotechnological tools for the design of neglected disease diagnostic serological assays [18–23, 32, 33]. The phage display through specific antibodies and selected amino acid sequences can be identical or present physicochemical characteristics or spatial organization similar enough to the original epitope to induce an immunoprotective response [34]. In the present study, which aimed to select peptides for* L. braziliensis *diagnosis infection, we selected 36 of 428 phage clones that showed recognition against antibodies from the patients' pool sera that exhibited high titer with* L. braziliensis* SAs, in ELISA test. Three peptides were synthetized and hamsters were immunized with them. Antibodies from animals recognized proteins from* L. braziliensis* SAs. After that, we have shown that the mimotopes can identify human cutaneous leishmaniosis on ELISA approach. The peptides have induced antibodies that recognized 52–76 kDa protein in a SDS-PAGE assay. Analyzing the mimotopes' sequence, it was verified that possibly we are dealing with the GP-63 protein which is a* Leishmania* spp. surface protease that is involved in parasite virulence and host cell interaction [35, 36]. GP-63 is a glycoprotein that represents 1% of the total of the promastigotes and is crucial for evasion of host cells from immune response. It is responsible for inactivating the lysosomes enzymes and influences the CD4+ T-cells lymphocyte activity by reducing the cell-mediated immune response [37, 38]. In addition, it cleaves intracellular peptides, preventing the presentation of antigens and inhibiting the chemotaxis of macrophages [39]. Many researches have been conducted seeking to develop vaccines against leishmaniasis using the GP-63 [40–43].

In the second step of our work, all peptides, individually or in MIX, recognized antibodies against* Leishmania *in patient serum with cutaneous leishmaniosis. When tested in immunodiagnosis by ELISA assay, the P1 antigen showed the best sensitivity/specificity. In general, P1 and MIX showed the best results (64 to 72% and 72 to 79% sensitivity for groups G1 and G2, resp.) between the peptides here tested. The ELISA test using SA of* L. braziliensis* promastigote showed sensitivities of 80 to 91% in the analysis of patients of G1 (with parasites isolated) and G2 (only with clinical diagnosis). These different values may be related to the panel-evaluated sera with procedures performed during the production of antigens, such as the incubation time of the reagent, the blocking solutions that were used, and the types of plates that were used [44]. Variations in the studied population can also create differences in sensitivity profile and the specificity of the tests. In patients with recent injuries (1 to 6 months of evolution), frequent serologic negativity and greater sensitivity to parasitological testing have been detected [3]. Another concern regarding the use of SA in ELISA is the existence of different strains of* Leishmania *behavior in different culture media. Studies show that expression can occur from different antigenic epitopes when the parasites are grown in media with different formulations [45, 46].

The results obtained here with the peptides are of extreme importance considering the need to produce a new ELISA test for the diagnosis of CL and the scarcity of studies that have investigated this form of the disease. The ELISA test offers advantages in terms of its application for immunodiagnosis of leishmaniosis. This is because it has versatility: its results are not being dependent on the observer in the culture and can be adapted for automation, allowing its use on a large scale. Obtaining purified antigens for the diagnosis of infectious and parasitic diseases is promising because it provides reproducibility of the tests. These molecules are stable and do not depend on specific means for their production cultivation.

Most studies concerning the detection of leishmaniosis have investigated the diagnosis of the canine visceral leishmaniosis form of the disease. These studies reveal that the sensitivities and specificities found using synthetic peptides or recombinant proteins in ELISA were, respectively, 75 and 90% [12], 88 and 95% [47], 76 to 100% and 90 to 97% [15], and 100 and 98% [48]. As for human VL sensitivity and specificity, the figures stand at 100 and 100% [49], 81 and 10% [50], and 76 to 100% and 90 to 97% [15].

Analyzing the cross-reactivity,* Trypanosoma cruzi* is the agent with the highest reactivity in all antigens used. This can be explained by the phylogenetic relationship between* T. cruzi* and* Leishmania* [51]. This cross-reaction is more important in the visceral leishmaniasis form, given the clinical signs of the disease. The cross-reactivity was resolved when it was used with the MIX of the peptides. This information is of paramount importance, since only immunogenic molecules can generate specific antibodies. Since these three peptides have this property, they could prove useful as immunization tools and for use in other immunological strategies.

New researches highly recommend testing these antigens using other immunological methodologies, for example, multiplex ELISA that has the advantage of allowing simultaneous analysis of multiple peptides. Otherwise, in regions with few resources, immunochromatographic analyses or skin test could be performed like the Montenegro Test. In addition, the peptides obtained in this study show similarity with sequences of virulence factors associated with* Leishmania* spp. The next step will be to isolate the protein and make the sequencing. It can be postulated that prior inoculation of these virulence factors could disrupt the course of a future infection, and thus these molecules could be used in immunoprophylactic strategies.

## Figures and Tables

**Table 1:** Enrichment of anti-*L. braziliensis* IgG binding phage after four rounds of biopanning. The phages bound are expressed as the ratio of the phage output titer and the phage input titer.

Biopanning cycles	Phages added (CFU)	Phages eluted (CFU)	% of bound phages (×10^−2^)	Enrichment
1	2.9 · 10^11^	3.6 · 10^5^	0.012	
2	2.0 · 10^11^	3,8 · 10^7^	1.900	158.3x
3	2.0 · 10^11^	6.6 · 10^7^	3.300	275x
4	2.0 · 10^11^	9.8 · 10^7^	4.900	408.3x

**Table 2:** Peptide sequences obtained after performing the phage display technique using immunoglobulins G specific to *Leishmania braziliensis*. The researches in GenBank were made using as microorganisms *L. braziliensis.*

Peptide 1	G	H	R	M	P	P	T	S	V	S	A	L	A	R	P	NCBI reference sequence
GP63		:	:										:			XP_001562922.1
	$\underset{59}{Q}$	Q	H	R	**P**	**P**	G	**S**	**V**	**S**	**A**	**L**	G	L	$\underset{73}{P}$	

Peptide 2	T	M	V	P	K	E	P	N	P	L	S	G	L	R	K	

GP63		:					:			:			:			XP_001562922.1
	$\underset{204}{A}$	S	**V**	**P**	S	**E**	**P**	G	V	**L**	A	T	A	V	$\underset{218}{I}$	

Peptide 3	S	K	P	Q	P	N	N	F	K	L	N	S	L	G	S	

GP63		:		:		:	:	:	:	:				:		XP_001562922.1
	$\underset{276}{S}$	N	L	R	G	R	D	Y	E	V	P	V	**L**	S	$\underset{290}{S}$	

The numbering below the alignment refers to the amino acid position in the protein sequence.

Bold letters denote identical amino acid residues.

The colon symbol (:) indicates amino acid residues with similar properties. Among the properties that can be shared between amino acids are hydrophobicity, polarity, charge, and presence of aromatic rings.

**Table 3:** Reactivity of peptides (P1, P2, and P3 and MIX) and soluble antigen (SA) against sera from patients with Chagas disease, leprosy, and tuberculosis. The cut-off was provided by the ROC curve.

Antigen	Cut-off	Chagas disease	Leprosy	Tuberculosis
P1	0.351	0/10	1/10	3/10
P2	0.327	1/10	2/10	1/10
P3	0.235	2/10	2/10	3/10
MIX	0.192	0/10	1/10	2/10
AS	0.101	6/10	2/10	0/10

MIX: P1 + P2 + P3; SA: soluble antigen of *Leishmania braziliensis*.

## References

[1] Desjeux P. (2004). Leishmaniasis: current situation and new perspectives. *Comparative Immunology, Microbiology and Infectious Diseases*.

[2] Alvar J., Vélez I. D., Bern C. (2012). Leishmaniasis worldwide and global estimates of its incidence. *PLoS ONE*.

[3] Szargiki R., de Castro E. A., Luz E., Kowalthuk W., Machado Â. M., Thomaz-Soccol V. (2009). Comparison of serological and parasitological methods for cutaneous leishmaniasis diagnosis in the state of Paraná, Brazil. *Brazilian Journal of Infectious Diseases*.

[4] de Paiva-Cavalcanti M., de Morais R. C. S., Pessoa-e-Silva R. (2015). Leishmaniases diagnosis: an update on the use of immunological and molecular tools. *Cell and Bioscience*.

[5] Faber W. R., Oskam L., Van Gool T. (2003). Value of diagnostic techniques for cutaneous leishmaniasis. *Journal of the American Academy of Dermatology*.

[6] Nuzum E., White F., Thakur C. (1995). Diagnosis of symptomatic visceral leishmaniasis by use of the polymerase chain reaction on patient blood. *Journal of Infectious Diseases*.

[7] Le Fichoux Y., Quaranta J.-F., Aufeuvre J.-P. (1999). Occurrence of *Leishmania infantum* parasitemia in asymptomatic blood donors living in an area of endemicity in southern France. *Journal of Clinical Microbiology*.

[8] Lachaud L., Dereure J., Chabbert E. (2000). Optimized PCR using patient blood samples for diagnosis and follow-up of visceral leishmaniasis, with special reference to AIDS patients. *Journal of Clinical Microbiology*.

[9] Montenegro J. (1926). Cutaneous reaction in leishmaniasis. *Archives of Dermatology and Syphilology*.

[10] Yoneyama K. A. G., de Peder L. D., Lonardoni M. V. C., Silveira T. G. V. (2007). Diagnosis of American cutaneous leishmaniasis by enzyme immunoassay in patients from Northern Paraná State, Brazil. *Brazilian Journal of Infectious Diseases*.

[11] Menezes-Souza D., de Oliveira Mendes T. A., de Araújo Leão A. C., de Souza Gomes M., Fujiwara R. T., Bartholomeu D. C. (2015). Linear B-cell epitope mapping of MAPK3 and MAPK4 from *Leishmania braziliensis*: implications for the serodiagnosis of human and canine leishmaniasis. *Applied Microbiology and Biotechnology*.

[12] Chávez-Fumagalli M. A., Martins V. T., Testasicca M. C. S. (2013). Sensitive and specific serodiagnosis of *Leishmania infantum* infection in dogs by using peptides selected from hypothetical proteins identified by an immunoproteomic approach. *Clinical and Vaccine Immunology: CVI*.

[13] Seyed N., Zahedifard F., Safaiyan S. (2011). *In silico* analysis of six known *Leishmania major* antigens and *in vitro* evaluation of specific epitopes eliciting HLA-A2 restricted CD8 T cell response. *PLoS Neglected Tropical Diseases*.

[14] Duarte A., Queiroz A. T. L., Tosta R. (2015). Prediction of CD8^+^ Epitopes in *Leishmania braziliensis* Proteins Using EPIBOT: *in silico* search and *in vivo* validation. *PLoS ONE*.

[15] Oliveira G. G. S., Magalhães F. B., Teixeira M. C. A. (2011). Characterization of novel *Leishmania infantum* recombinant proteins encoded by genes from five families with distinct capacities for serodiagnosis of canine and human visceral leishmaniasis. *American Journal of Tropical Medicine and Hygiene*.

[16] Kumar S., Kumar D., Chakravarty J., Rai M., Sundar S. (2012). Identification and characterization of a novel *Leishmania donovani* antigen for serodiagnosis of visceral leishmaniasis. *American Journal of Tropical Medicine and Hygiene*.

[17] Parmley S. F., Smith G. P. (1988). Antibody-selectable filamentous fd phage vectors: affinity purification of target genes. *Gene*.

[18] Capelli-Peixoto J., Chávez-Olórtegui C., Chaves-Moreira D. (2011). Evaluation of the protective potential of a *Taenia solium* cysticercus mimotope on murine cysticercosis. *Vaccine*.

[19] Alban S. M., de Moura J. F., Minozzo J. C., Mira M. T., Soccol V. T. (2013). Identification of mimotopes of *Mycobacterium leprae* as potential diagnostic reagents. *BMC Infectious Diseases*.

[20] Alban S. M., De Moura J. F., Thomaz-Soccol V. (2014). Phage display and synthetic peptides as promising biotechnological tools for the serological diagnosis of leprosy. *PLoS ONE*.

[21] Fogaça R. L., Capelli-Peixoto J., Yamanaka I. B. (2014). Phage-displayed peptides as capture antigens in an innovative assay for *Taenia saginata*-infected cattle. *Applied Microbiology and Biotechnology*.

[22] Toledo-Machado C. M., Bueno L. L., Menezes-Souza D. (2015). Use of Phage Display technology in development of canine visceral leishmaniasis vaccine using synthetic peptide trapped in sphingomyelin/cholesterol liposomes. *Parasites and Vectors*.

[23] Autran B., Carcelain G., Combadiere B., Debre P. (2004). Therapeutic vaccines for chronic infections. *Science*.

[24] Vélez I. D., Gilchrist K., Martínez S. (2009). Safety and immunogenicity of a defined vaccine for the prevention of cutaneous leishmaniasis. *Vaccine*.

[25] Lowry O. H., Rosebrough N. J., Farr A. L., Randall R. J. (1951). Protein measurement with the Folin phenol reagent. *The Journal of Biological Chemistry*.

[26] Ribas-Silva R. C., Navasconi T. R., de Souza Braga L. (2015). Serological and molecular investigation of cutaneous leishmaniasis in healthy individuals from an American Cutaneous Leishmaniasis-endemic region. *American Journal of Infectious Diseases*.

[27] Harlow E., Lane D. (1998). *Antibodies: A Laboratory Manual*.

[28] Bradford M. M. (1976). A rapid and sensitive method for the quantitation of microgram quantities of protein utilizing the principle of protein-dye binding. *Analytical Biochemistry*.

[29] Bonnycastle L. L. C., Mehroke J. S., Rashed M., Gong X., Scott J. K. (1996). Probing the basis of antibody reactivity with a panel of constrained peptide libraries displayed by filamentous phage. *Journal of Molecular Biology*.

[30] Metz C. E. (1978). Basic principles of ROC analysis. *Seminars in Nuclear Medicine*.

[31] Zweig M. H., Campbell G. (1993). Receiver-operating characteristic (ROC) plots: a fundamental evaluation tool in clinical medicine. *Clinical Chemistry*.

[32] Thomaz-Soccol V., Alban S. M., Moura J. F. Uso de peptídeos miméticos de *Mycobacterium leprae* para diagnóstico e vacinas.

[33] Thomaz-Soccol V., Alban S. M., Seger J. Peptídeos miméticos de *Leishmania* sp. Processo para sua obtenção e aplicações.

[34] de Moura J., Alvarenga L. M., Thomaz-Soccol V. (2017). Biotechnological role of phage-displayed peptides for the diagnosis of neglected tropical diseases. *Human and Animal Health Applications*.

[35] Etges R., Bouvier J., Bordier C. (1986). The major surface protein of *Leishmania* promastigotes is a protease. *Journal of Biological Chemistry*.

[36] Joshi P. B., Kelly B. L., Kamhawi S., Sacks D. L., McMaster W. R. (2002). Targeted gene deletion in *Leishmania major* identifies leishmanolysin (GP63) as a virulence factor. *Molecular and Biochemical Parasitology*.

[37] Gupta G., Oghumu S., Satoskar A. R. (2013). Mechanisms of immune evasion in leishmaniasis. *Advances in Applied Microbiology*.

[38] Hey A. S., Theander T. G., Hviid L., Hazrati S. M., Kemp M., Kharazmi A. (1994). The major surface glycoprotein (gp63) from *Leishmania major* and* Leishmania donovani* cleaves CD4 molecules on human T cells. *Journal of Immunology*.

[39] Garcia M. R., Graham S., Harris R. A., Beverley S. M., Kaye P. M. (1997). Epitope cleavage by *Leishmania* endopeptidase(s) limits the efficiency of the exogenous pathway of major histocompatibility complex class I-associated antigen presentation. *European Journal of Immunology*.

[40] Connell N. D., Medina-Acosta E., Mcmaster W. R., Bloom B. R., Russell D. G. (1993). Effective immunization against cutaneous leishmaniasis with recombinant bacille Calmette-Guérin expressing the *Leishmania* surface proteinase gp63. *Proceedings of the National Academy of Sciences of the United States of America*.

[41] Habibi G. R., Khamesipour A., McMaster W. R., Mahboudi F. (2001). Cytokine gene expression in healing and non-healing cases of cutaneous leishmaniasis in response to *in vitro* stimulation with recombinant gp63 using semi-quantitative RT-PCR. *Scandinavian Journal of Immunology*.

[42] Jaafari M. R., Ghafarian A., Farrokh-Gisour A. (2006). Immune response and protection assay of recombinant major surface glycoprotein of Leishmania (rgp63) reconstituted with liposomes in BALB/c mice. *Vaccine*.

[43] Bhowmick S., Ravindran R., Ali N. (2008). Gp63 in stable cationic liposomes confers sustained vaccine immunity to susceptible BALB/c mice infected with *Leishmania donovani*. *Infection and Immunity*.

[44] Reithinger R., Dujardin J.-C., Louzir H., Pirmez C., Alexander B., Brooker S. (2007). Cutaneous leishmaniasis. *Lancet Infectious Diseases*.

[45] Schuster F. L., Sullivan J. J. (2002). Cultivation of clinically significant hemoflagellates. *Clinical Microbiology Reviews*.

[46] Somanna A., Mundodi V., Gedamu L. (2002). In vitro cultivation and characterization of *Leishmania chagasi* amastigote-like forms. *Acta Tropica*.

[47] Faria A. R., Costa M. M., Giusta M. S. (2011). High-throughput analysis of synthetic peptides for the immunodiagnosis of canine visceral leishmaniasis. *PLoS Neglected Tropical Diseases*.

[48] Porrozzi R., Santos Da Costa M. V., Teva A. (2007). Comparative evaluation of enzyme-linked immunosorbent assays based on crude and recombinant leishmanial antigens for serodiagnosis of symptomatic and asymptomatic Leishmania infantum visceral infections in dogs. *Clinical and Vaccine Immunology*.

[49] Passos S., Carvalho L. P., Orge G. (2005). Recombinant *Leishmania* antigens for serodiagnosis of visceral leishmaniasis. *Clinical and Diagnostic Laboratory Immunology*.

[50] Costa M. M., Penido M., Santos M. S. (2012). Improved canine and human visceral leishmaniasis immunodiagnosis using combinations of synthetic peptides in enzyme-linked immunosorbent assay. *PLoS Neglected Tropical Diseases*.

[51] Badaró R., Benson D., Eulálio M. C. (1996). rK39: A cloned antigen of *Leishmania chagasi* that predicts active visceral leishmaniasis. *The Journal of Infectious Diseases*.

